
JOURNAL INFORMATION
==============================
NLM Title Abbreviation: ScientificWorldJournal
Journal ID: ScientificWorldJournal
NLM Journal ID: 101131163
Journal ID: TSWJ

The Scientific World Journal

ISSN: 2356-6140
EISSN: 1537-744X
Publisher: Wiley

ARTICLE INFORMATION
==============================
PMCID: PMC6084520
PMID: 30147538
DOI: 10.1100/TSW.2001.105
Article ID: 602837
Article version: 1
Subjects: Short Report

Functional Decline in Human Neutrophils with Age

Butcher Stephen *
Chahal Hema
Savey Elizabeth
Killampalli V. V.
Alpar E. K.
Lord Janet M.

*Stephen Butcher: s.k.butcher.20@bham.ac.uk
Publication date: 2001
Electronic publication date: 2001 Nov 30
Volume: 1
First page: 67
Last page: 67
Copyright: Copyright © 2001 Janet, M. Lord et al.
Copyright year: 2001
License: This is an open access article distributed under the Creative Commons Attribution License, which permits unrestricted use, distribution, and reproduction in any medium, provided the original work is properly cited.
License URL: https://creativecommons.org/licenses/by/3.0/

Funding: Biotechnology and Biological Sciences Research Council

==============================
Miami *Nature Biotechnology* Short Reports
*TheScientificWorld* (2001) 1(S3), 67SR
ISSN 1532–2246; DOI 10.1100/TSW.2001.105

### FUNCTIONAL DECLINE IN HUMAN NEUTROPHILS WITH AGE

Stephen Butcher*, Hema Chahal, Elizabeth Savey, V.V. Killampalli, E.K.Alpar, and Janet M. Lord
* S.K.Butcher.20@bham.ac.uk

**INTRODUCTION.** The neutrophil forms the first line of defence against rapidly dividing bacteria and fungi. As humans age they become more susceptible to infection by these microbes (1). We have investigated the neutrophil functions affected by ageing in humans and examined the underlying mechanisms resulting in the age related decline in function. We have observed a reduction in cell surface expression of the Fc receptor CD16, accompanied by a decrease in the phagocytic ability of neutrophils from elderly individuals. In elderly patients with neutrophilia, in which the fraction of blood neutrophils recently released from the bone marrow is increased, CD16 was not significantly increased. These data suggest that in the elderly neutrophils are released from the bone marrow with compromised function, indicative of a defect in progenitor cell production.

**METHODS.** Neutrophils were isolated from the peripheral blood of healthy young (18–35 yrs), healthy old (>65 yrs) volunteers and from elderly patients with neutrophilia induced by bacterial infection or trauma. Cell surface expression of CD11b and CD16 was assessed by flow cytometry and apoptosis was measured by annexin V binding. Superoxide response to fMLP was assessed by the lucinogen chemoluminescent assay (2). Phagocytosis of opsonised yeast was determined by light microscopy and phagocytosis of FITC labelled E coli (Orpegen) was determined by flow cytometry. Serum cytokine levels were analysed by ELISA (R&D Systems).

**RESULTS.** Neutrophils from healthy young and old individuals were analysed for their ability to phagocytose opsonised yeast and *E. coli* and to generate superoxide in response to the bacterial product fMLP. Whilst the killing mechanism of superoxide generation was not decreased, the ability of neutrophils from elderly volunteers to phagocytose yeast and bacteria was significantly decreased. Phagocytosis is mediated via the ligation of receptors for complement (CD11b) and the Fc portion of immunoglobulin (CD16). Expression of CD16 was significantly decreased ($p<0.01$) in neutrophils from the elderly, with no loss of CD11b. Loss of CD16 has been reported to be a marker for apoptosis in neutrophils (3), but analysis of annexin V binding revealed that the neutrophils from the elderly group were not increased in their level of apoptosis or the rate at which they entered apoptosis in culture. Loss of CD16 on neutrophils can be effected GM-CSF, but our data showed that serum GM-CSF levels were not raised in the elderly. To determine whether newly produced neutrophils were already deficient in CD16 we have examined CD16 expression in neutrophils from elderly patients with neutrophilia caused by bacterial infection or trauma. In both situations CD16 was not increased, suggesting that in the elderly neutrophils are released from the bone marrow with reduced CD16 expression.

**DISCUSSION.** Our data show that neutrophils from elderly humans have a significant reduction in expression of the Fc receptor CD16, accompanied by a reduction in phagocytic ability, which could in part explain the decline in neutrophil function observed in the elderly. Interestingly, the superoxide response of neutrophils to fMLP was not reduced in the elderly and in fact was significantly increased, an indication of neutrophil priming. Priming is known to delay neutrophil apoptosis. Reduced CD16 is also an effect produced by the priming cytokine GM-CSF. However, we did not find altered apoptosis in neutrophils from elderly volunteers and GM-CSF serum levels were not affected by age. The reduced CD16 of neutrophils from the elderly would therefore not appear to be related to a modulation of apoptosis or to the influence of GM-CSF. An alternative explanation is that neutrophils are released from the bone marrow in a CD16 deficient state. We therefore examined CD16 expression on neutrophils isolated from elderly patients with neutrophilia. Neutrophils are short-lived cells (half life 8-12h) and during periods of infection or trauma their production is vastly increased, leading to an increase in freshly released neutrophils in the blood. CD16 expression was not significantly increased during neutrophilia, suggesting that CD16 was already reduced when the neutrophils were released into the circulation. We are now attempting to determine whether neutrophil progenitors have reduced CD16 *per se*, or whether GM-CSF levels are raised in bone marrow in the elderly giving the reduced expression of CD16 in neutrophils released from the marrow to the blood.

**ACKNOWLEDGEMENTS.** Funded by the BBSRC SAGE initiative.

**REFERENCES.**

1. Finelstein, M.S., Petkun, W.M., and Freedman, M.L. (1983) J. Am. Geriatr. Soc. 31, 68-74
2. Robinson, P., Wakefield, D., Briet, S., Easter, J., and Penny, R. (1984) Infect. Immun. 43, 744-752
3. Hart, S.P., Ross, J.A., Ross, K., Haslett, C., and Dransfield, I. (2000) Cell Death Differ. 7, 493-503
